# ﻿First record of the genus *Sceloattalus* Wittmer, 1966 (Coleoptera, Malachiidae) from China, with description of a new species

**DOI:** 10.3897/zookeys.1181.107115

**Published:** 2023-09-27

**Authors:** Junbo Tong, Sergei E. Tshernyshev, Haoyu Liu, Yuxia Yang

**Affiliations:** 1 The Key Laboratory of Zoological Systematics and Application, School of Life Science, Institute of Life Science and Green Development, Hebei University, Baoding, 071002, China Hebei University Baoding China; 2 Institute of Systematics and Ecology of Animals, Siberian Branch of the Russian Academy of Sciences, Frunze Street 11, Novosibirsk, 630091, Russia Institute of Systematics and Ecology of Animals, Siberian Branch of the Russian Academy of Sciences Novosibirsk Russia; 3 Tomsk State University, Lenina prospekt 36, Tomsk, 634050, Russia Tomsk State University Tomsk Russia

**Keywords:** Cleroidea, melyrid lineage, new faunistic record, taxonomy

## Abstract

The genus *Sceloattalus* Wittmer, 1966 is recorded from China for the first time. A new species, *S.nigroprominens* Tong & Yang, **sp. nov.**, is described and illustrated from Xizang Autonomous Region of China. This new species is the fourth member of *Sceloattalus* and can be easily distinguished from others by the yellow antennae with a small black spot at apex of last antennomere, black elytra without metallic lustre, all tarsi yellow, and hind tibiae yellow to black. The generic diagnosis of *Sceloattalus* is resummarized. An identification key to all known species of *Sceloattalus* is also updated.

## ﻿Introduction

*Sceloattalus* Wittmer, 1966 is a small genus within the tribe Attalini of the family Malachiidae. It consists of three species restricted to Nepal and West Bengal region of India (Tshernyshev 2015). Wittmer (1966) and Tshernyshev (2015, 2021) have previously contributed to the taxonomy of this genus.

*Sceloattalus* can be easily distinguished from all other genera of Attalini by the sexually dimorphic hind tibiae, which are strongly dilated on the outer side in males, but simple in females (Wittmer 1966; Tshernyshev 2015). Interestingly, *Sceloattalus* looks similar to *Sceloebaeus* Tshernyshev, 2015 of the tribe Ebaeini in having modified hind tibiae, but *Sceloattalus* can be distinguished from *Sceloebaeus* by the following characters: body larger (4.6–6.5 mm in length), pronotum yellow, tarsomere 4 shorter than 3, tarsomere 2 of the fore tarsi with spur-like comb in males, and undivided pygidium (apical tergite) and ultimate abdominal ventrite (apical sternite). In contrast, *Sceloebaeus* has a smaller body (about 2.5 mm), a black pronotum, tarsomere 4 approximately as long as 3, simple tarsi lacking specialized male structures, and a divided pygidium and ultimate abdominal ventrite (Wittmer 1999; Tshernyshev 2015).

In this study, a new species of *Sceloattalus* is described from Xizang Autonomous Region. *Sceloattalus* is recorded from China for the first time, and it represents the fifth genus belonging to the tribe Attalini in the Chinese fauna (Mayor 2007; Tong et al. 2022). The new species is described and illustrated here under the name of *S.nigroprominens* sp. nov., and with this addition to the genus, the generic diagnosis is necessarily resummarized here. Additionally, an identification key to all known species of *Sceloattalus* is updated.

## ﻿Materials and methods

In this study, malachiid beetles are considered as a family, Malachiidae (Majer 1994, 2002; Mayor 2007; Bocakova et al. 2012; Constantin 2021), instead of a subfamily (e.g. Gimmel et al. 2019).

For descriptions, specialized male structures and genitalia were studied. The term “specialized male structures” is not analogous to the term “Excitatoren”; the latter term means different kinds of structures located in different parts of the males of soft-winged flower beetles and bearing ducts of pheromone glands necessary for female attraction and successful copulation (Evers 1956, 1963, 1988; Matthes 1962). The “specialized male structures” include all typical parts of the male, irrespective of their having pheromone glands or not.

Terminology of terminalia morphology is according to Lawrence et al. (2010), namely (in comparison with previously used terms) pygidium for apical tergite, ultimate abdominal ventrite for apical sternite, and endophallus for the inner sac of the aedeagus.

For dissection, the specimen had its abdomen detached and soaked in 10% solution of sodium hydroxide (NaOH) by boiling for several minutes. The ovipositor was dyed with haematoxylin. Genitalia were dissected, cleaned, and transferred to glycerol on slides, then photographed with a LEICA DFC450 digital camera attached to a LEICA M205 A microscope. LAS V.4.7 software was used to capture genitalia images. External morphology was observed with a Nikon SMZ1500 stereomicroscope. Images of adults were taken with a Canon EOS 80D digital camera and focus stacked in Helicon Focus 7. The final plates were prepared in Adobe Photoshop CS 6.0.

## ﻿Taxonomy

**Family Malachiidae Leach, 1815**

### ﻿Genus Sceloattalus

Taxon classificationAnimaliaColeopteraMelyridae

Wittmer, 1966

51717191-9A99-56BF-BD91-0013CB120934

Sceloattalus Wittmer, 1966: 236. Type species: Sceloattalusnepalensis Wittmer, 1966, by original designation.

#### Diagnosis.

**Figure 1.**
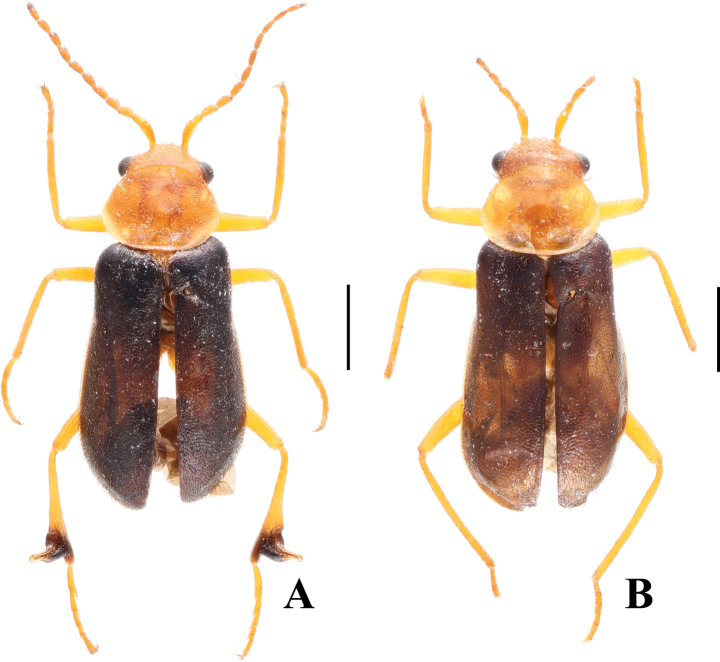
Habitus of *Sceloattalusnigroprominens* Tong & Yang, sp. nov., dorsal views **A** holotype, male **B** paratype, female. Scale bars: 1.0 mm.

**Figure 2.**
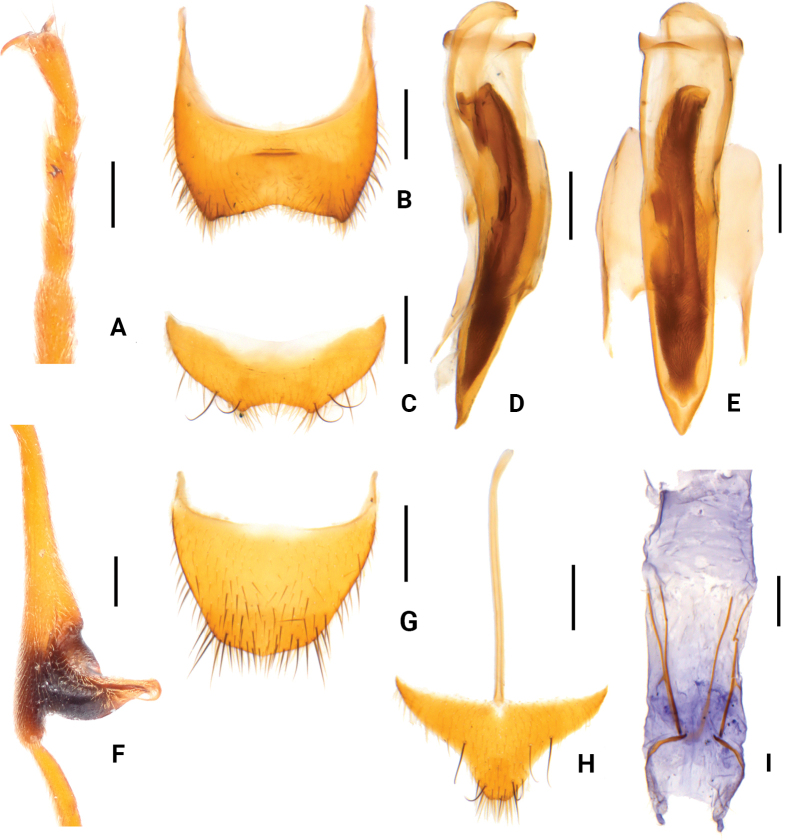
*Sceloattalusnigroprominens* Tong & Yang, sp. nov. **A–F**, holotype, male **A** fore tarsi **B** pygidium (apical tergite) **C** ultimate abdominal ventrite (apical sternite) **D** male genitalia, lateral view **E** male genitalia, ventral view **F** hind tibia **G–I** paratype, female **G** pygidium (apical tergite) **H** ultimate abdominal ventrite (apical sternite) **I** ovipositor. Scale bars: 0.2 mm.

Body medium-sized to large, 4.6–6.5 mm in length. Pronotum uniformly yellow, elytra black usually with blue-metallic lustre, except for *S.nigroprominens* sp. nov. (Fig. 1A, B). Antennae simple and slender. Elytra lacking specialized structures. Male hind tibiae strongly dilated on outer side, and tarsomere 2 of fore tarsi produced in a spur-like comb; both simple in female (Fig. 2A, F). Ultimate abdominal ventrite (apical sternite) short and transverse with a terminal emargination (Fig. 2C).

#### Distribution.

China (Xizang), Nepal, India (West Bengal).

### ﻿ Sceloattalus nigroprominens

Taxon classificationAnimaliaColeopteraMelyridae

Tong & Yang sp. nov.

4A50673D-B55C-59DD-9C82-389816C2DE01

https://zoobank.org/06931FC1-7002-4050-9AD6-97A5270FAD95

Figs 1 , 2

#### Diagnosis.

This species is similar to *Sceloattaluskopetzi* Tshernyshev, 2015 but can be distinguished from the latter by the antennae mostly yellow with a small black spot at apex of last antennomere, elytra without metallic lustre, and tarsi of all legs yellow. In contrast, *S.kopetzi* has antennae mostly black with antennomeres 1–4 yellow, elytra with blue-metallic lustre, and tarsi yellow to black.

#### Materials examined.

***Holotype*: China: Xizang**: ♂, Nang, Nyingchi, 3100 m elev., 29.VI.1997, Chaodong Zhu leg (IZAS). ***Paratype*: China: Xizang**: 1♀, same data as holotype (IZAS). The type specimens are deposited in Institute of Zoology, Chinese Academy of Sciences, Beijing, China (IZAS).

#### Description.

**Male.** Length of body 4.8 mm; width at widest part of elytra 1.9 mm and at the base of elytra 1.5 mm.

Body yellow, with elytra and metathorax black. (Fig. 1A). Mandibles somewhat black. Antennae with a small black spot at apex of last antennomere. Fore legs with a small black spur-like comb on tarsomere 2 (Fig. 2A), hind legs yellow to black. Vesicles and thoracic mesepimera yellow. Elytra covered with black setae, while head and pronotum double consisting of yellow adpressed pubescence and sparse black stiff bristles. Sculptures evenly punctuated, stronger on elytra than other parts.

Head narrower than pronotum. Frons slightly impressed. Clypeus distinct. Antennae filiform extending over the apical quarter of the elytra; antennomere 1 elongate and clavate; 2 shortened and rectangular; 3 and 4 slightly widened; 5–11 cylindrical and approximately equal in length.

Pronotum transverse, anterior margin slightly protruding, posterior margin flattened and curved upwards.

Scutellar shield small, triangular, almost completely covered by pronotum.

Elytra slightly widened behind base; base of elytra approximately as wide as pronotum. Humeri distinct, slightly protruding. Disc without metallic lustre. Elytral apices evenly rounded.

Hind wings normally developed.

Legs slender. Hind femora not reaching elytral apices. All tibiae thin and straight. All tarsi with 5 tarsomeres; tarsomere 2 of fore tarsi produced in a spur-like comb, not extending over the tarsomere 3 (Fig. 2A); tarsomere 1 longest and tarsomere 4 shortest in mid and hind legs. Claws thin, curved, with membrane at base and long setae at middle.

Metathorax simple, lacking appendages. Pygidium (apical tergite) broad, with triangular emargination on distal side (Fig. 2B); ultimate abdominal ventrite (apical sternite) short, transverse, with a triangular emargination terminally (Fig. 2C). Tegmen short and wide; aedeagus stout, slightly narrowing to blunt apex in ventral view (Fig. 2D, E).

**Female.** Length of body 4.8 mm; width at widest part of elytra 1.9 mm and at the base of elytra 1.5 mm.

Similar to male species except for hind legs uniformly yellow and not dilated at apices of tibiae (Fig. 1B). Pygidium subtrapezoid (Fig. 2G). Ultimate abdominal ventrite subtriangular, with long spiculum ventrale (Fig. 2H). Ovipositor elongate and membranous (Fig. 2I).

#### Distribution.

China (Xizang).

#### Etymology.

The specific epithet *nigroprominens* derives from the Latin words “*niger*” (black) and “*prominens*” (prominent) and refers to the black prominence on hind tibiae.

### ﻿Key to the species of the genus *Sceloattalus* Wittmer, 1966

Males only

**Table:** 

1	Head uniformly black; all legs black	***S.nepalensis* Wittmer, 1966**
–	Head mostly yellow; legs uniformly or somewhat yellow	**2**
2	Antennae yellow with a small black spot at apex of last antennomere; elytra without metallic lustre; fore legs uniformly yellow	***S.nigroprominens* Tong & Yang, sp. nov.**
–	Antennae mostly black; elytra with blue or violet metallic lustre; fore legs yellow to black	**3**
3	Antennae black with antennomeres 1–4 yellow; tarsi yellow to black; mid and hind legs almost completely yellow	***S.kopetzi* Tshernyshev, 2015**
–	Antennae black with outer sides of antennomeres 1–3 yellow; tarsi uniformly black; mid and hind legs almost completely black	***S.bengalensis* (Pic, 1907)**

## Supplementary Material

XML Treatment for Sceloattalus

XML Treatment for Sceloattalus nigroprominens

## ﻿Additional information

### Author ORCIDs

Junbo Tong https://orcid.org/0000-0002-0116-0204

Sergei E. Tshernyshev https://orcid.org/0000-0001-5931-9241

Haoyu Liu https://orcid.org/0000-0003-1383-5560

Yuxia Yang https://orcid.org/0000-0002-3118-6659

### Data availability

All of the data that support the findings of this study are available in the main text.
